# Exploring Fully Navigated Minimally Invasive Spine Surgery-Transforaminal Lumbar Interbody Fusion (MISS-TLIF): An Institutional Analysis

**DOI:** 10.7759/cureus.93260

**Published:** 2025-09-26

**Authors:** Ajay Krishnan, Sandesh Subhash Agarawal, Mahesh Sagar, Bharat R Dave, Shivanand C Mayi, Ravi Ranjan Rai, Preety Krishnan, Mirant B Dave, Amritesh Singh, Mikeson Panthackel, Arjit Vashishtha

**Affiliations:** 1 Spine Surgery, Stavya Spine Hospital and Research Institute, Ahmedabad, IND; 2 Radiology, Stavya Spine Hospital and Research Institute, Ahmedabad, IND; 3 Orthopedics, University College of Medical Sciences, Guru Teg Bahadur Hospital, Delhi, IND

**Keywords:** 3d ct o-arm navigation, mis-tlif, percutaneous pedicle screw, proximal facet joint violation, workflow

## Abstract

Introduction

Traditionally, percutaneous pedicle screw fixation has been guided using a C-arm. In recent years, 3D CT-based navigation systems have enabled improved visualization and navigated cage placement. However, the sequence of guide wire insertion, screw placement, and cage insertion continues to vary among surgeons, often depending on individual preference. Such variability can disrupt workflow efficiency and underutilize the full potential of navigation technology. A standardized, stepwise approach, initiated with a preoperative 3D O-arm spin and followed by sequential navigation at each step, can optimize workflow and minimize radiation exposure.

Materials and methods

We conducted a retrospective analysis of 44 patients who underwent fully navigated minimally invasive spine surgery-transforaminal lumbar interbody fusion (MISS-TLIF). After a 3D O-arm spin, a single 1-inch skin incision was made at the projected convergence points of the screws, and guide wires were inserted. On the decompression side, a fascial incision through the same skin opening allowed access via a medialized transmuscular TLIF approach. The procedure was performed using loupe magnification, a microscope, or an exoscope, depending on the case. Cage insertion was navigated, and pedicle screws were then placed over the pre-positioned guide wires. Final fixation was completed after confirming placement with a postoperative 3D spin. We analyzed patient demographics, pathology, surgical details, and radiological parameters. Outcomes included pedicle screw perforation, proximal facet joint violation (PFJV), local disc angle, posterior disc height, and pedicle screw convergence angle.

Results

Of the 44 patients included, 35 were female and nine were male. Navigated screw and cage placement was successfully achieved in all cases. The mean estimated blood loss (EBL) was 100 mL, and the average operative time was 124 ± 23.4 minutes. Visualization techniques included loupe magnification (n = 35), operating microscope (n = 9), and exoscope (n = 3). The Oswestry Disability Index demonstrated significant improvement at a mean follow-up of 38.7 ± 13.44 months. A total of 176 pedicle screws were placed, with 164 positioned accurately. PFJV occurred in 7% of screws (2% Grade 2 and 5% Grade 1 breaches). Radiological outcomes, including local disc angle and posterior disc height, were satisfactory and consistent with existing literature. Pedicle screw trajectory demonstrated improved medial convergence.

Conclusions

Fully navigated 3D CT-guided MISS-TLIF is a streamlined procedure that maximizes the capabilities of navigation technology while minimizing workflow disruptions. It overcomes the limitations of conventional C-arm-based techniques and variable surgeon-dependent workflows. This method enhances surgical precision, reduces complications such as pedicle screw breaches and facet joint violations, and facilitates improved screw convergence and cage placement accuracy.

## Introduction

Lumbar spinal fusion is one of the most widely performed surgical procedures for degenerative spinal disorders, instability, deformity, and trauma. Over the past several decades, fusion techniques have undergone significant refinement, driven by the dual goals of improving clinical outcomes and minimizing surgical morbidity. Traditional open approaches, although effective in achieving fusion and decompression, are associated with extensive muscle dissection, greater blood loss, prolonged hospitalization, and delayed functional recovery. These limitations prompted the development of minimally invasive spine surgery (MISS), which, over the past two decades, has revolutionized the surgical management of lumbar pathologies by reducing perioperative morbidity while preserving the efficacy of conventional open procedures [1,2].

Within MISS, transforaminal lumbar interbody fusion (TLIF) has gained widespread acceptance as a reliable technique that allows circumferential fusion through a unilateral corridor with minimal disruption of the posterior tension band. Percutaneous pedicle screw fixation (PPSF) remains the cornerstone of minimally invasive lumbar fusion. Conventionally, PPSF is performed under C-arm fluoroscopic guidance, which, while effective, poses challenges such as a steep learning curve, reliance on multiple fluoroscopic shots to ensure accuracy, and cumulative radiation exposure to both patients and the surgical team [3,4]. Furthermore, fluoroscopy provides only two-dimensional imaging, limiting visualization of anatomical variations and increasing the risk of screw malposition.

The advent of intraoperative 3D CT-based navigation systems has significantly transformed spinal instrumentation. Navigation has been shown to improve pedicle screw placement accuracy, reduce revision rates, and enhance safety margins, particularly in anatomically complex or revision cases [5]. When integrated into minimally invasive workflows, navigation further optimizes surgical precision by providing real-time anatomical feedback and enabling instrument tracking within the operative field.

Despite these advantages, variability persists in how navigation is applied in lumbar fusion workflows. The sequence of guidewire insertion, pedicle screw placement, and interbody cage positioning often depends heavily on surgeon preference and institutional protocols. This variability can reduce efficiency, increase reliance on additional intraoperative imaging, and limit the full potential of navigation technology. In particular, navigation is frequently restricted to pedicle screw placement, with interbody cage insertion still performed under fluoroscopy, thereby constraining the benefits of a fully navigated procedure [6]. The absence of a standardized, stepwise approach reduces consistency, prolongs operative time, and may diminish the accuracy and safety advantages of navigation.

To address these challenges, there is a clear need to establish and validate a comprehensive, standardized workflow that maximizes navigation use throughout all stages of MISS-TLIF. Ideally, such a protocol would begin with a single intraoperative 3D O-arm spin, followed by navigated facetectomy, endplate preparation, interbody cage placement, and pedicle screw insertion, streamlining workflow, minimizing redundant imaging, and fully leveraging the benefits of navigation technology.

The primary objective of this retrospective analysis of 44 patients was to evaluate the safety, accuracy, and clinical and radiological outcomes of a standardized, fully navigated MISS-TLIF workflow utilizing 3D O-arm navigation. The secondary objective was to assess workflow efficiency. We implemented and examined a stepwise protocol that integrates navigation at every stage, beginning with a single 3D O-arm acquisition and concluding with final implant placement.

## Materials and methods

This was a retrospective observational study conducted at a single tertiary spine center. A total of 44 consecutive patients underwent fully navigated MISS-TLIF between January 2020 and December 2023. The inclusion criteria were adult patients diagnosed with degenerative lumbar spine pathologies, such as instability-related low back pain (ILBP), non-instability low back pain (NILBP), radiculopathy due to lateral recess stenosis (LRS), and central canal stenosis, who underwent single-level MISS-TLIF using a fully 3D CT navigation-guided technique. The exclusion criteria included multilevel fusion, prior lumbar instrumentation, spinal tumors, infections, or trauma. All patients underwent a detailed clinical examination, along with standard radiographs, MRI scans, and CT scans as required. A preoperative 3D O-arm spin was performed to facilitate surgical navigation and to plan screw trajectories and cage placement.

Surgical technique

**Figure 1:**
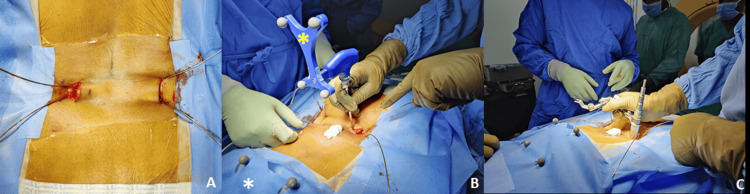
(A) Intraoperative image of an L4-5 MISS-TLIF, showing guidewires placed in a convergent manner using 3D CT O-arm navigation. (B) A medialized fascial incision was made through the same 1-inch skin incision, and a navigated PAK needle was employed to determine the precise trajectory (yellow asterisk) for decompression and fusion. (C) The tubular retractor was then docked onto the predetermined, navigation-guided guidewire.

Following general anesthesia and prone positioning, a reference frame was attached to the skin, and a 3D CT scan was obtained using the O-arm. A 1-inch longitudinal skin incision was made bilaterally at the projected convergence points of the pedicle screws. Guidewires were inserted under navigational guidance (Figure 1A).

On the decompression side, a fascial incision was made through the same skin opening to allow a medialized transmuscular TLIF approach using tubular retractors (Figure 1B, 1C). Decompression was performed using loupe magnification (n = 35), an operating microscope (n = 9), or an exoscope (n = 3), depending on case logistics and availability. After adequate decompression, discectomy and endplate preparation were performed.

**Figure 2:**
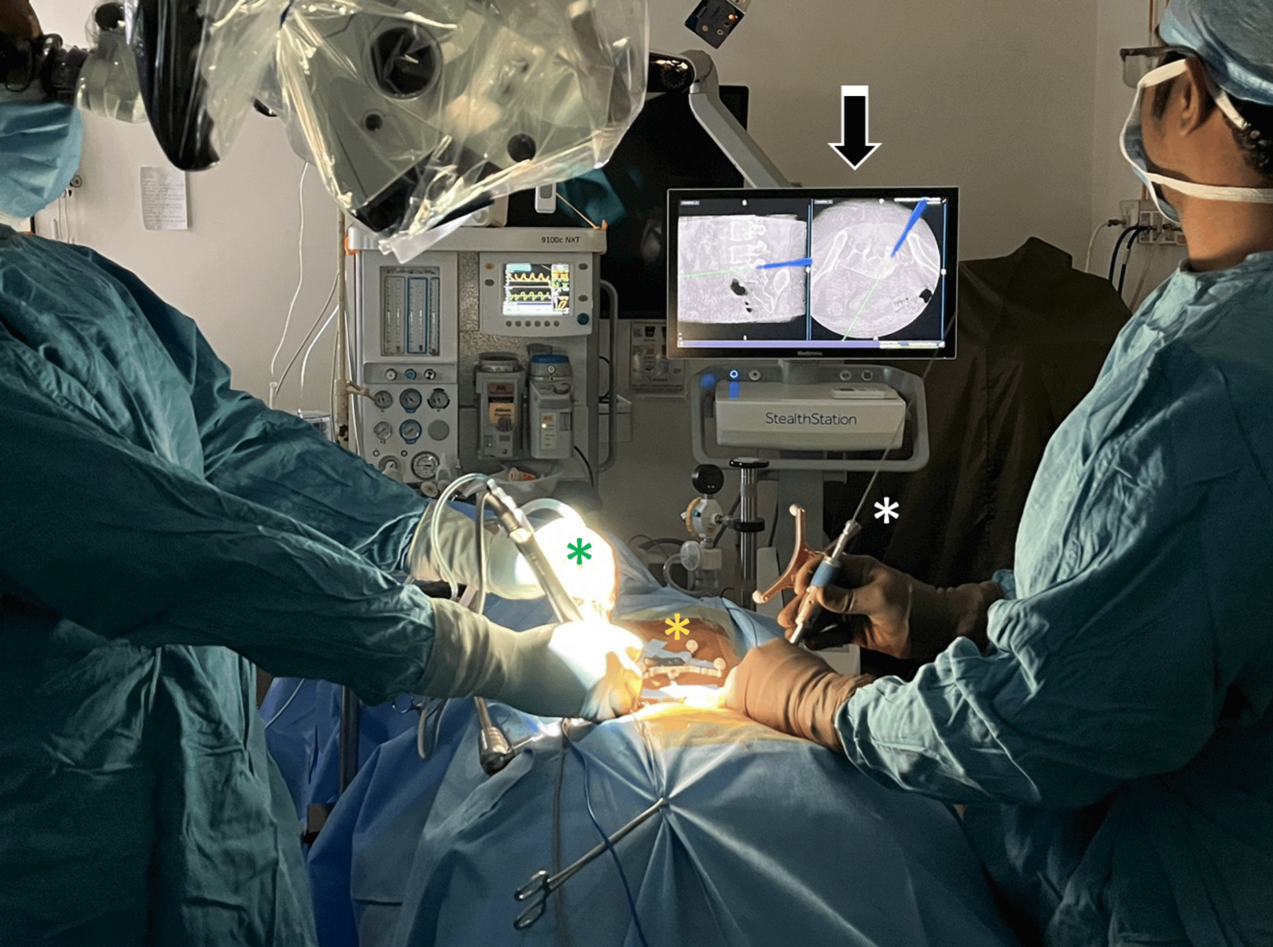
Operative setup of a single-level fully navigated MISS-TLIF. The left-sided surgeon is operating through the tubular retractor under an operating microscope. The reference frame is fixed to the skin (yellow asterisk). A Misonix bone scalpel is being used for bony work through the tubular retractor (green asterisk). The second surgeon expedites workflow by inserting contralateral pedicle screws on pre-positioned guidewires (white asterisk) under O-arm navigated stealth guidance (black arrow). This represents an upgraded, time-saving workflow, different from the one reported in this study (in the current study, guidewires were inserted first, with screw placement performed only after decompression and navigated interbody cage insertion). This progressive protocol allows for a more efficient, unhindered workflow and improved team synergy.

**Figure 3:**
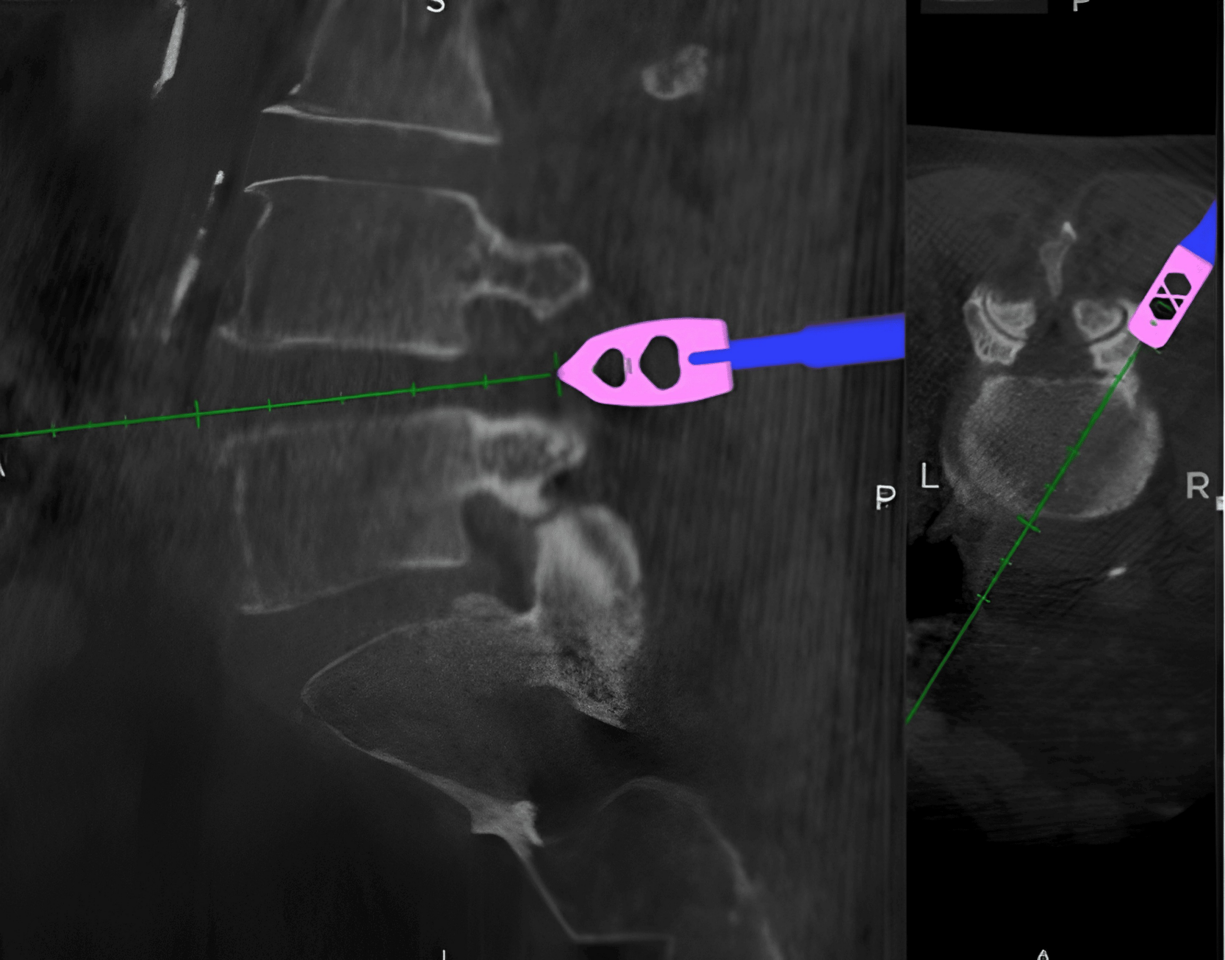
Navigated precise placement of a predetermined interbody cage.

To expedite workflow, a second surgeon placed the contralateral pedicle screw construct on pre-positioned guidewires under O-arm navigated stealth guidance (Figure 2). Interbody cage insertion was also navigated, with various cage types used (Adaptix, PEEK, or titanium) (Figure 3). Pedicle screws were then inserted over the pre-positioned guidewires, and final fixation was confirmed using a 3D O-arm spin. 

Clinical outcomes were assessed using the Oswestry Disability Index (ODI) [7], the Numerical Rating Scale (NRS) [8] for back and leg pain, and the Medical Research Council (MRC) grading [9] for muscle strength. These evaluations were conducted preoperatively, at three months, at two years, and at final follow-up.

Radiological assessments included measurement of the local disc angle (°) and posterior disc height (mm), both preoperatively and postoperatively, to evaluate segmental alignment correction and adequacy of decompression. Additional radiological parameters included pedicle screw trajectory angles, assessment of medial shaft breaches within all four quadrants of the vertebral pedicle cross-section [10], and grading of proximal facet joint violation (PFJV) [11] on postoperative imaging.

All radiological measurements were performed using validated RadiAnt DICOM Viewer software, with assessor blinding by non-operating surgeons to minimize bias. Fusion was inferred based on the absence of implant loosening, preservation of spinal alignment, and sustained clinical improvement; however, no dedicated postoperative CT scan was obtained to confirm arthrodesis.

Statistical analysis

Statistical analyses were performed using IBM SPSS Statistics for Windows, Version 3.0 (Released 2025; IBM Corp., Armonk, NY, USA). Continuous variables were expressed as mean ± SD. The one-way ANOVA test was used to compare ODI [7] and NRS [8] scores across multiple time points. A paired t-test was used for pre- and postoperative comparisons of MRC [9] scores. A p-value < 0.05 was considered statistically significant.

Assumptions for these tests were verified. Normality of data distribution was assessed using the Shapiro-Wilk test and visually confirmed with Q-Q plots, confirming approximate normal distribution. Homogeneity of variances was tested using Levene’s test. Independence of observations was ensured by the study design.

## Results

The mean age of the study participants was 47.5 ± 10.9 years. Among them, 16 (36.3%) were in the 41-50-year age group, followed by 10 (22.7%) in the 31-40-year age group. Only two participants were younger than 30 years. Of the total, nine (20.4%) were male and 35 (79.5%) were female (Table 1). The mean height was 153.1 cm, the mean weight was 65.4 kg, and the mean BMI was 26.8 kg/m². Comorbidities included osteoporosis in 13 (29.5%), hypertension in 10 (22.7%), obesity in 8 (18.2%), and diabetes mellitus in 2 (4.5%) participants.

**Table 1:** Demographic distribution of affected individuals

Variable	Frequency/mean ± SD
Age group (years)	
21-30	2 (4.5%)
31-40	10 (22.7%)
41-50	16 (36.3%)
51-60	11 (25%)
>60	5 (11.3%)
Mean age	47.5 ± 10.9 years
Gender	
Male	9 (20.4%)
Female	35 (79.5%)
Anthropometric measurements	
Height (cm)	153.1
Weight (kg)	65.4
BMI (kg/m²)	26.8
Comorbidities	
Osteoporosis	13 (29.5%)
Hypertension	10 (22.7%)
Diabetes mellitus	2 (4.5%)
Obesity	8 (18.18%)
Hypothyroidism	1 (2.2%)
Ischemic heart disease	1 (2.2%)
Asthma	0 (0%)
Others	4 (9%)

**Figure 4:**
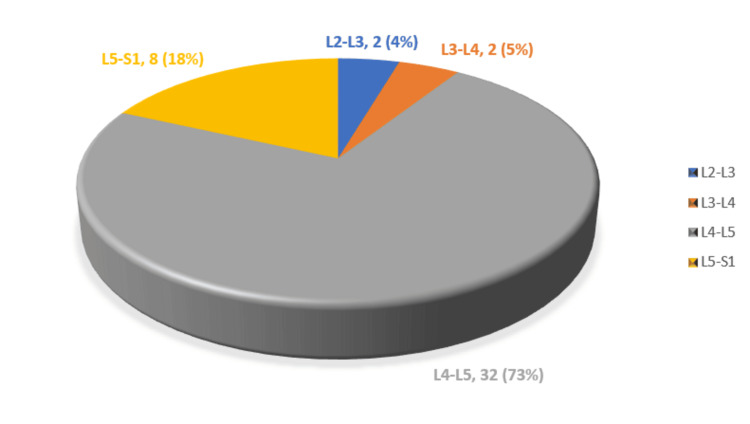
Level of operation among study participants (n = 44)

Among the cases, two patients had ILBP and 22 had NILBP. No substantial intraoperative or postoperative complications were noted. The most commonly affected level was L4-L5, observed in 32 cases (72.7%), followed by other levels (Figure 4).

The mean ODI score [7] demonstrated significant improvement, decreasing from a preoperative mean of 38.6 ± 5.08 to 12.5 ± 5.3 at the final follow-up (p < 0.001, ANOVA). Similarly, NRS [8] scores for back and leg pain significantly improved from 8.6 ± 0.85 and 8.65 ± 0.83 preoperatively to 0.79 ± 0.9 and 0.81 ± 0.75, respectively, at the final follow-up (p < 0.001, ANOVA). Assumption checks confirmed the appropriateness of ANOVA and paired t-tests, supporting the robustness and reliability of the findings. However, there was no statistically significant difference in mean MRC muscle strength scores [9] between preoperative (4.95 ± 0.21) and postoperative (4.95 ± 0.21) assessments (p = 0.413, paired t-test) (Table 2).

**Table 2:** Functional outcomes Values are presented as mean ± SD. Preoperative and final follow-up values were compared using paired t-tests. Test statistics (t-values) were calculated using approximate standard error based on reported means and SDs. MRC, Medical Research Council; NRS, Numerical Rating Scale; ODI, Oswestry Disability Index

Outcome measure	Pre-op (mean ± SD)	Final follow-up (mean ± SD)	Statistical test	Test statistic	p-Value
ODI score [7]	38.6 ± 5.08	12.5 ± 5.3	Paired t-test	t = 23.6	<0.001
NRS back pain [8]	8.6 ± 0.85	0.79 ± 0.9	Paired t-test	t = 35.3	<0.001
NRS leg pain [8]	8.65 ± 0.83	0.81 ± 0.75	Paired t-test	t = 36.1	<0.001
MRC score [9]	4.95 ± 0.21	5.0 ± 0.1	Paired t-test	t = 1.43	0.16

The mean operative time was 124 ± 23.4 minutes (range: 75-180 minutes). The EBL was 100 ± 39.5 mL (range: 45-250 mL). Among the 44 cases, angular instability was observed in 30 patients, while facet effusion and lysis were present in 18 and 17 cases, respectively.

All surgeries were performed under navigation guidance. Visualization techniques included loupe magnification in 35 cases, an operating microscope in nine cases, and an exoscope in three cases. Although visualization methods varied in magnification and field of view, no direct outcome comparisons were made. In terms of decompression, nine patients underwent over-the-top plus (OTT+) decompression, while 35 underwent standard LRS decompression. Interbody cage selection included Adaptix cages in 15 patients, poly-ether-ether-ketone (PEEK) cages in 13 patients, and titanium cages in 16 patients.

Radiological evaluation demonstrated significant postoperative improvement in spinal alignment and disc restoration. The mean local disc angle increased from 7.1 ± 2.6° preoperatively to 10.5 ± 2.1° postoperatively (p = 0.002), indicating effective segmental lordosis correction. The mean posterior disc height improved from 7.5 ± 0.80 mm to 13.2 ± 0.83 mm (p = 0.001), reflecting adequate disc space distraction and decompression.

Medial shaft screw breach [10] was minimal across all quadrants: 0.15 ± 0.30 mm (right upper), 0.06 ± 0.25 mm (left upper), 0.09 ± 0.29 mm (right lower), and 0.13 ± 0.30 mm (left lower), all within safe limits. Screw trajectories were consistent, with mean angles of 33.21 ± 2.2°, 33.13 ± 2.4°, 33.80 ± 2.5°, and 32.50 ± 2.4° in the respective quadrants. PFJV [11] occurred in 7% of screws (2% Grade 2, 5% Grade 1 breaches).

Fusion was inferred in all cases based on maintained reconstruction, absence of correction loss, and lack of implant loosening at a mean follow-up of 38.7 months. No postoperative CT scans were performed to confirm arthrodesis.

## Discussion

Despite its benefits, MISS-TLIF presents several technical challenges, including a steep learning curve, limited working space, and reduced tactile feedback, which may contribute to complications such as cage malposition or dural injury. To overcome these challenges, the use of intraoperative navigation and 3D imaging has become increasingly common. Kulkarni et al. demonstrated that multimodal 3D navigation significantly enhances the precision of MISS-TLIF, thereby reducing complications and radiation exposure [12]. Similarly, Liu et al. reported improved surgical accuracy and deformity correction in elderly patients with low-grade spondylolisthesis using 3D navigation [13].

In our study, the mean patient age was 47.5 ± 10.9 years (range: 27-74 years), with a predominance of female patients (79.5%). Compared to the cohort in the study by Kim et al., which featured an older and more male-dominant population with a similar body mass index, our patients were younger and predominantly female [14]. Functional outcomes demonstrated statistically significant improvements in ODI and NRS scores from the preoperative period to the final follow-up (p < 0.05). However, MRC grading showed no significant change, indicating stable motor function postoperatively. These findings are consistent with those of Phan et al., who reported significant reductions in disability scores along with a 100% radiographic fusion rate at 12 months [15]. Min and Yoo also observed sustained functional improvements during long-term follow-up after MISS-TLIF [16].

In our series of 44 patients, the mean operative time was 124 ± 23.4 minutes, and the mean EBL was 100 ± 39.5 mL. Both values were lower than those reported by Phan et al. [15], suggesting that our approach may be more efficient and less invasive.

A systematic review by Weiss et al. highlighted that although MISS-TLIF reduces overall complication rates compared to open surgery, specific risks such as dural tears, neural injury, and hardware malposition remain relevant [17]. The use of intraoperative navigation in our study likely contributed to the low incidence of these complications, improving pedicle screw accuracy and minimizing the risk of iatrogenic injury. This reflects the broader trend in spine surgery, which emphasizes not only clinical outcomes but also safety and precision.

Compared to MISS-TLIF, open TLIF is associated with greater paraspinal muscle injury and a higher incidence of facet joint violation (FJV). Babu et al. reported that FJV occurred more frequently with open techniques (46.2%) than with percutaneous pedicle screw placement (15.2%) [18]. In a comparative study, Uotani et al. demonstrated significantly lower pedicle screw breach rates with 3D navigation-guided MISS-TLIF (one of 30) compared with fluoroscopy-guided techniques (four of 13), along with better cage orientation in the navigation group (p = 0.024) [19]. Nilssen et al. similarly reported fewer rostral FJVs with navigation (4.3%) compared to robotic-assisted screw placement (15.4%) [20]. In contrast, Singhatanadgige et al. reported FJV rates as high as 10.9%, whereas our study demonstrated a lower incidence of proximal FJV (7%) along with minimal medial screw shaft breach across all quadrants (≤0.15 mm), supporting the superior accuracy and safety of our technique [21].

The workflow in MISS-TLIF using 3D CT O-arm navigation plays a crucial role in improving surgical safety, accuracy, and efficiency. A consistent, well-structured workflow facilitates surgeon training and reproducibility, leading to more predictable outcomes [22]. Although technological integration reportedly improves results, detailed stepwise surgical protocols outlining workflow optimization and time-saving strategies remain limited.

The limitations of this study include its retrospective design, heterogeneity of the patient group, and relatively small sample size (n = 44). Fusion was inferred clinically and radiographically based on implant stability, spinal alignment, and functional outcomes, without CT confirmation. While this approach is consistent with similar studies, it limits the strength of conclusions regarding arthrodesis. Meta-analyses comparing MISS-TLIF and open TLIF have reported no significant differences in fusion rates, supporting the reliability of clinical and radiographic evaluation when CT is not routinely performed [23].

Other limitations include the absence of a comparator group, restricting conclusions on superiority; the single-surgeon design, which may limit generalizability; and the lack of interobserver reliability testing for radiological measurements, potentially introducing bias. The use of varied visualization techniques (loupe magnification, operating microscope, and exoscope) could also act as confounding factors. Furthermore, cost and accessibility issues related to advanced navigation systems may limit broader adoption.

Another important consideration is the reduced reliance on tactile feedback when using advanced navigation systems. While these technologies enable younger surgeons to rapidly acquire technical skills, there is a risk of bypassing fundamental surgical principles and bailout techniques, which could have medicolegal implications. Although our workflow appears efficient, prospective comparative studies are required to establish its true advantages.

Workflow comparison studies between robotic and navigation systems remain scarce due to challenges in standardization and ethical concerns. Finally, as this was a single-surgeon study (AK), generalizability is limited by individual technique and preference. The primary objective of this study was to assess functional and radiological outcomes following fully navigated MISS-TLIF. Workflow efficiency was considered qualitatively, but objective quantification was not feasible.

## Conclusions

This study demonstrates that fully navigated MISS-TLIF is a feasible and safe option for degenerative lumbar spine disorders, improving pedicle screw accuracy while reducing FJV and medial screw shaft breach. Given the absence of a comparator group, our findings primarily establish feasibility and favorable outcomes rather than superiority. Future studies should focus on developing well-defined stepwise workflows with detailed protocols that emphasize time efficiency, reduction of workflow hindrance, and reproducibility.
